# A DOG's View of Fanconi Anemia: Insights from *C. elegans*

**DOI:** 10.1155/2012/323721

**Published:** 2012-05-30

**Authors:** Martin Jones, Ann Rose

**Affiliations:** Department of Medical Genetics, University of British Columbia, Vancouver, BC, Canada V6T 1Z4

## Abstract

*C. elegans* provides an excellent model system for the study of the Fanconi Anemia (FA), one of the hallmarks of which is sensitivity to interstrand crosslinking agents. Central to our understanding of FA has been the investigation of DOG-1, the functional ortholog of the deadbox helicase *FANCJ*. Here we review the current understanding of the unique role of DOG-1 in maintaining stability of G-rich DNA in *C. elegans* and explore the question of why DOG-1 animals are crosslink sensitive. We propose a dynamic model in which noncovalently linked G-rich structures form and un-form in the presence of DOG-1. When DOG-1 is absent but crosslinking agents are present the G-rich structures are readily covalently crosslinked, resulting in increased crosslinks formation and thus giving increased crosslink sensitivity. In this interpretation DOG-1 is neither upstream nor downstream in the FA pathway, but works alongside it to limit the availability of crosslink substrates. This model reconciles the crosslink sensitivity observed in the absence of DOG-1 function with its unique role in maintaining G-Rich DNA and will help to formulate experiments to test this hypothesis.

## 1. Introduction

The helicase, FANCJ, is required for the Fanconi Anemia (FA) pathway to function properly and thus maintain genome integrity. In humans, *FANCJ* mutations have been identified in early-onset breast cancer patients [1, 2] and FA complementation group J patients [3–5]. However, the role of FANCJ in the FA pathway of DNA repair is not fully understood. Some insights have been gained from research on DOG-1 (*D*eletions *O*f *G*-rich DNA), the *Caenorhabditis elegans* functional ortholog of FANCJ [6–9]. However, even in this relatively simple model system, important questions remain. An outstanding issue is the relationship between the relatively well-known function of DOG-1/FANCJ in preventing replication blocks at unresolved secondary structures and its function in resistance to interstrand crosslinks (ICLs). Previous work from our group has shown that DOG-1 acts upstream of, or parallel to, FCD-2 in the maintenance of G-tracts [7] but is dispensable for FCD-2 focus formation in response to ICL generating agents [8]. One possibility is that DOG-1 takes on two different functions, one in G4 DNA resolution and one in FA crosslink repair. On the other hand, it is possible that its ability to unwind G-rich secondary structure may be sufficient to explain its role in both situations. Here we summarize the current understanding of DOG-1/FANCJ function and hypothesize how to reconcile the two known roles for this protein with its helicase function.

## 2. DOG-1 Is Required for Maintenance of G-Tracts

DOG-1 was discovered as being essential for the maintenance of G-rich DNA [6] and was subsequently shown to be the functional ortholog of FANCJ [8]. The value of *C. elegans* as a model for Fanconi Anemia and ICL repair has been thoroughly reviewed in Youds et al. [9]. An understanding of DOG-1's role in replication and repair began with the observation that it is a mutator. This was immediately recognizable in *C. elegans* because of the appearance of spontaneous morphological mutants (described in Cheung et al. [6]) and further explored by the capture and characterization of mutational changes in genes essential for survival (lethal mutations) maintained using a genetic balancer [10]. In *dog-1* mutants, the manifestation of the morphological *Vab* (*V*ariable *AB*normal) phenotype was linked to the gene *vab-1*. An examination of the molecular nature of the *vab-1* mutations revealed small deletions that were detectable by PCR. These deletions initiated at the 5′ end of poly-C or the 3′ end of poly-G stretches of DNA and extended for variable distances. These observations led to the proposal that the deletions were occurring as a result of structural blocks to lagging-strand synthesis [6]. In this model, poly-G stretches present in the *C. elegans* genome form secondary structures. These secondary structures require the helicase function of DOG-1 to resolve them, allowing fork progression. In the absence of the helicase function, deletions are formed between the stalled fork and the upstream Okazaki fragment initiation. Another research group subsequently confirmed the prediction that Okazaki-sized deletions occurred on the lagging strand by using unbiased array comparative hybridization (aCGH) of DOG-1-minus genomes [11].In this study, it was shown that deletions occurred exclusively at sequences that could form quadruplex structures (G4) at a frequency of 4% per site per animal generation. In the human genome, there are estimated to be >300,000 G4 forming sites [12], and these have potentially mutagenic properties implicated in development of cancer susceptibility in the absence of FANCJ function.

**Figure 1:**
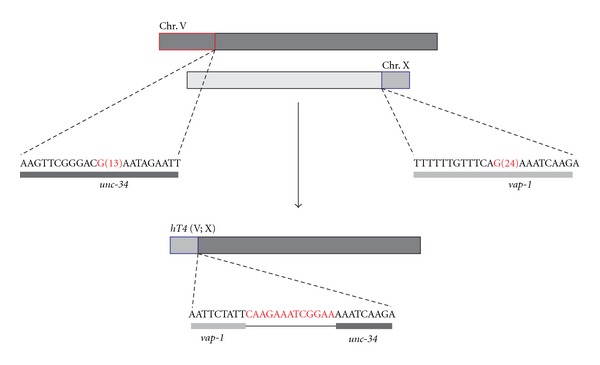
Schematic of the *dog-1*-derived translocation, *hT4 *(V; X). Sequences near the left end of chromosome V were deleted (red box), whereas the right end of chromosome X was duplicated (blue box). PCR primers were designed and used to determine the DNA sequence across the junction of V and X in *hT4 *(described in [10]).

Further work from our laboratory revealed that in the absence of DOG-1 large chromosomal rearrangements occurred [10]. The rearrangements included larger deletions, duplications of chromosomal fragments, and translocations between chromosomes, in addition to the small deletions detectable by PCR. These large rearrangements were identified because they acquired lethal mutations, which could be isolated and characterized with the use of a balancer chromosome that provided a rescuing wild-type allele in a stable genetic construct (reviewed in [13]). The analysis showed that 1% of the chromosomes acquired lethal lesions [10], giving a forward mutation frequency greater than tenfold of the spontaneous frequency. The frequency is equivalent to that for 500 Rads of ionizing radiation [14]. Rearrangements derived from *dog-1* mutant that were examined by aCGH revealed that in most (but not all) cases the breakpoints occurred in G-rich DNA. In one example, a translocation between chromosome V and the X-chromosome was formed. In this case, the right end of the X-chromosome was duplicated and attached to the left breakpoint of a deletion at left end of chromosome V (Figure 1). The breakpoint on chromosome V is in a 24 bp G/C tract, while the breakpoint on the X is in a “short” 13 bp G-rich sequence. In vertebrates, large rearrangements have also been observed in the absence of FANCJ function. In avian DT40 cell lines, large-scale genomic deletions occurred at the rearranged immunoglobulin heavy chain locus (IgH) in the absence of *FANCJ*, but not other FA genes [15]. These researchers found that in *FANCJ *mutant cells cultured for two months, G4 sequences detected by aCGH were found at the breakpoints of one deletion. However, not all breaks occurred in G-rich DNA, suggesting that other sequences are also susceptible to breakage in the absence of FANCJ.

## 3. Homologous Recombination and Translesion Synthesis Compensate for the Absence of DOG-1

Repair pathways that compensate for the absence of DOG-1 in *C. elegans* have been identified. These include homologous recombination (HR) repair and translesion synthesis (TLS), but not nonhomologous end joining (NHEJ) [7]. In human cell lines, monoubiquitylation of FANCD2 is followed by HR repair. Our genetic analysis has shown that DOG-1 mutants that are also mutant for FCD-2 (FANCD2) exacerbate G-tract deletions [8], as are the HR repair components, BRD-1 (BARD1), RAD-51 (RAD51), and XPF-1 (XPF). Similarly, DOG-1 mutants lacking the TLS polymerases, POL eta and POL kappa have significantly more PCR-detectable G-tract deletions than DOG-1 by itself. That the FA pathway and its downstream repair mechanisms are capable of resolving some G-tract-associated secondary structures in the absence of DOG-1 function indicates that the FA pathway is parallel to DOG-1, at least with respect to the maintenance of G-tracts. 

A recent study in DT40 cells has expanded the endogenous role of FANCJ. Recently, Sarkies et al. have shown that FANCJ coordinates two independent mechanisms to maintain epigenetic stability near G4 DNA motifs [16]. These mechanisms are dependent on the function of the Y-family polymerase REV1 and the helicases WRNs and BLMs. Similar epigenetic studies have not been performed in *C. elegans*. However, G-tract instability is significantly increased in DOG-1 mutants animals deficient in the BLM ortholog HIM-6 [7]. Mutants in the *C. elegans* WRNs ortholog WRN-1 do not exacerbate G-tract deletions, indicting that if a function in G-tract resolution is conserved in *C. elegans*, it is dependent on the presence of DOG-1. The *C. elegans* REV1 ortholog REV-1 has not been studied with respect to G-tract stability.

## 4. DOG-1 Functions to Reduce ICL-Induced Damage

A diagnostic feature of FA defects is the cross-link sensitivity of cultured cells. The presence of ICLs can result in error-prone repair leading to chromosomal instability (CIN) and cell death. In *C. elegans*, the absence of DOG-1 also results in sensitivity to ICL-inducing agents such as UVA-activated trimethylpsoralen, nitrogen mustard, and cisplatin, but not to X-rays or UVC [8]. Treatment of DOG-1-deficient animals with ICL agents can result in checkpoint-induced cell cycle arrest and apoptosis of germ cells, as well as chromatin bridges and breaks [8]. In response to ICL treatments, animal's doubly mutant for DOG-1 and FCD-2 are as equally sensitive as each of the single mutants, potentially placing the helicase function of DOG-1 in the same pathway as FCD-2 [8]. Furthermore, DOG-1 is not required for RAD-51 or FCD-2 foci formation after replication stress or ICL induction, possibly placing DOG-1 downstream of FCD-2. This data correlates with that reported by Bridge et al. [17] who demonstrated that *FANCJ* mutant DT40 cells are also not defective for FANCD2 focus formation.

**Figure 2:**
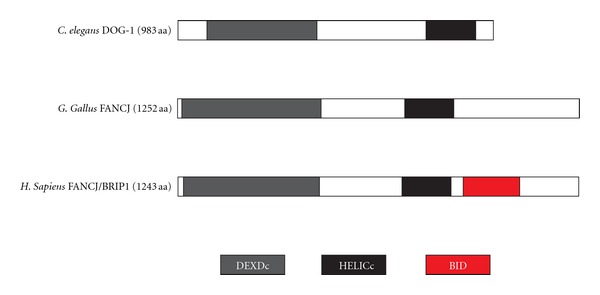
Protein schematic of FANCJ orthologs. *C. elegans* DOG-1, 983 aa, chicken FANCJ, 1252 aa and human, 1243 aa FANCJ proteins illustrating the position of the conserved DEAD Box (DEXDc) and helicase (HELICc) domains. The BRCA-1 interaction domain of human FANCJ is illustrated (BID). A full protein sequence alignment of DOG-1 and human FANCJ is shown in [8].

In human cell lines, monoubiquitinlation of FANCD2 is followed by HR repair. During S phase, ICLs can block replication; consequently, HR and TLS are required to stabilize the fork and restart replication (reviewed in [18]). In *C. elegans*, HR repair alleviates the loss of DOG-1. DOG-1 does not function directly in DSB repair, however, as it is not sensitive to radiation-induced DSBs [8]. Bridge et al. determined that FANCJs role in ICL repair is independent of BRCA1 function by demonstrating rescue of *FANCJ* phenotypes in DT40 cells with the expression of human FANCJ/BRIP1 lacking its BRCA1-interaction domain [17]. Since DOG-1, like the avian FANCJ, does not contain the BRCA-1 interaction domain found in human FANCJ (Figure 2), we infer that the helicase function of DOG-1 is not required for HR-mediated DSB repair following replication block or ICL induction.

The type of repair pathway recruited following replication block is important in maintaining genome stability. In *C. elegans* [8] and in human and chicken cells [19], FA proteins regulate the decision to repair double strand breaks (DSBs) resulting from replication blocks or ICLs using error-free HR repair rather than error-prone nonhomologous end joining (NHEJ). In the Adamo et al. study [20], it was shown that FA-deficient human cell lines and *C. elegans* mutants had chromosomal abnormalities similar to those found in cell lines from cancer and FA patients. However, when the NHEJ component LIG-4 (LIG4) is lacking, the abnormalities do not occur. HR-mediated repair is proposed to be favored due to single-stranded DNA produced by FANCD2 [19]. In *C. elegans*, this result provides a potential inroad to further dissection of the role of FA in DNA repair and the maintenance of genome stability.

The relationship between TLS and HR repair in *C. elegans* has been teased apart somewhat by the characterization of two genes, *polq-1* (*POLQ*) and *hel-308* (*HELQ*) [21]. POLQ-1 has a helicase domain at the N-terminus and a polymerase domain at the C-terminus and has been implicated in recombination-independent and TLS-dependent ICL repair (reviewed in [22]). The helicase HEL-308, on the other hand, is proposed to function in HR along with the FA pathway in ICL repair. In *C. eleg*ans, there are two genetically distinct pathways, a BRC-1-POLQ-1 pathway and an FA (FCD-2, DOG-1)-HR-HEL-308 pathway. At least one of these pathways must be functional for animals survival as mutants in *hel-308* results in synthetic lethality when combined with *brc-1* mutants (reviewed in [9]). These results separate the helicase function of DOG-1 from the BRC-1/BRCA-1 repair pathway and further distinguish the role of DOG-1 as independent of HR repair. Initially these results may appear paradoxical. FCD-2 is not required for G-tract stability and the double mutant *dog-1*;* fcd-2* increased G-tract deletions 3-fold [8], placing DOG-1 upstream of the FA pathway. However, in the case of ICL sensitivity, the double mutant is not more sensitive. One interpretation of these data is that DOG-1 is epistatic to the FA pathway. Both findings are consistent with DOG-1 attempting unsuccessfully to remove the cross-linked structure.

How does this inform our understanding of DOG-1's helicase function and the relationship between G-rich secondary structures and ICLs? There is ample evidence that DOG-1 is unique in its role to maintain G-rich DNA that can form G4-like secondary structures [7, 8, 11]. Additionally, it has been demonstrated that purified FANCJ efficiently unwinds a variety of G4 structures dependent upon intrinsic FANCJ ATP hydrolysis and the availability of a 5′ ssDNA tail [22]. None of the other helicases that are able to unwind G4 structures can compensate for the loss of DOG-1. This is supported by the fact that in *C. elegans* DOG-1 has a unique phenotype and that in other systems only FANCJ has been shown to prevent breaks in G-rich DNA. These structures are, however, not covalently linked. There is no evidence that the DOG-1/FANCJ helicase can resolve covalently linked ICLs. So what is the connection?

**Figure 3:**
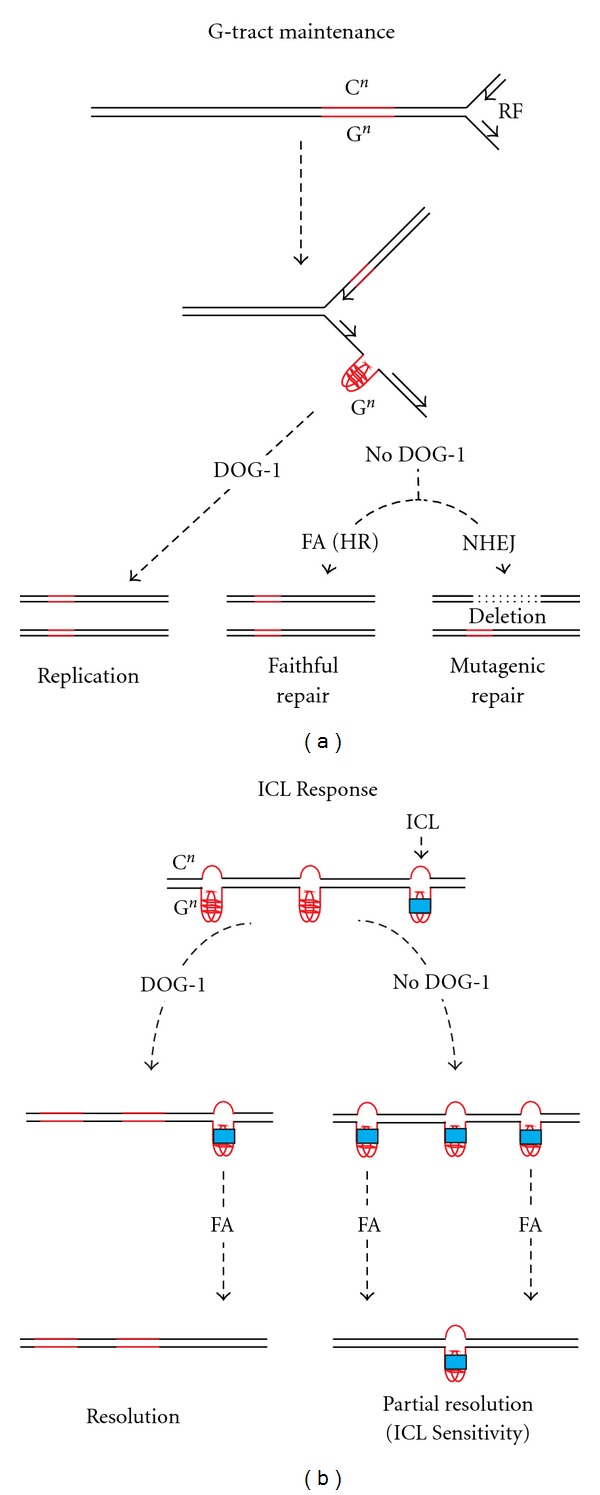
A model for DOG-1 function in genome stability and ICL response. The left panel illustrates DOG-1's role in G-tract maintenance. G4 formation on the lagging strand is resolved by the helicase function of DOG-1 and replication proceeds efficiently. In the absence of DOG-1 HR mediated by the FA pathway resolves a subset of stalled forks. Repair utilizing the mutagenic NHEJ repair mechanism results in deletions. The right panel describes a possible model for DOG-1 ICL sensitivity. In the presence of DOG-1, G4 structures may be resolved and not available as substrate for ICL stabilization. In the absence of DOG-1 G4 structures are available as substrate for ICL stabilization leading to an in increase in fork stalling, which is interpreted as an ICL sensitivity phenotype.

We propose the following model as a resolution of this apparent paradox (Figure 3). G4 structures are known to form in a variety of circumstances as proposed by Wu et al. [22], which could include within a single strand of DNA, between DNA strands and between strands on separate chromosomes. The latter resulting in chromosomal translocations if not repaired correctly. In the absence of crosslinking agents, these secondary structures can form and unform depending upon the availability of DOG-1. In the *C. elegans* genome, there are nearly 400 poly-G regions distributed along each of the chromosomes and this pattern of distribution is conserved in a related nematode [23] providing a rich source of substrate for DOG-1. In the presence of a crosslinking agent, many of which have affinity for G's, secondary structures formed by these G-rich regions might be targets for covalent crosslinking. Here we suggest that once the secondary structures are detected by FA pathway components the first responder is DOG-1. The pathway detector may not distinguish between a noncovalent secondary structure and a crosslink. If the structure is not covalently linked, DOG-1 resolves it. If it is covalently linked, and not resolved by DOG-1, FA pathway-directed TLS and HR repair the lesion. In the absence of DOG-1, there is likely to be an increase in stabilized G-rich structures that may be beyond the ability of the FA pathway to respond to, giving the appearance of a crosslink sensitive phenotype. Further experiments will be needed to move towards a more complete understanding of the crosstalk among FA proteins.

## References

[1] Cantor SB, Bell DW, Ganesan S (2001). BACH1, a novel helicase-like protein, interacts directly with BRCA1 and contributes to its DNA repair function. *Cell*.

[2] Seal S, Thompson D, Renwick A (2006). Truncating mutations in the Fanconi anemia J gene BRIP1 are low-penetrance breast cancer susceptibility alleles. *Nature Genetics*.

[3] Levitus M, Waisfisz Q, Godthelp BC (2005). The DNA helicase BRIP1 is defective in Fanconi anemia complementation group J. *Nature Genetics*.

[4] Levran O, Attwooll C, Henry RT (2005). The BRCA1-interacting helicase BRIP1 is deficient in Fanconi anemia. *Nature Genetics*.

[5] Litman R, Peng M, Jin Z (2005). BACH1 is critical for homologous recombination and appears to be the Fanconi anemia gene product FANCJ. *Cancer Cell*.

[6] Cheung I, Schertzer M, Rose A, Lansdorp PM (2002). Disruption of dog-1 in *Caenorhabditis elegans* triggers deletions upstream of guanine-rich DNA. *Nature Genetics*.

[7] Youds JL, O’Neil NJ, Rose AM (2006). Homologous recombination is required for genome stability in the absence of DOG-1 in *Caenorhabditis elegans*. *Genetics*.

[8] Youds JL, Barber LJ, Ward JD (2008). DOG-1 is the *Caenorhabditis elegans* BRIP1/FANCJ homologue and functions in interstrand cross-link repair. *Molecular and Cellular Biology*.

[9] Youds JL, Barber LJ, Boulton SJ (2009). *C. elegans*: a model of Fanconi anemia and ICL repair. *Mutation Research*.

[10] Zhao Y, Tarailo-Graovac M, O’Neil NJ, Rose AM (2008). Spectrum of mutational events in the absence of DOG-1/FANCJ in *Caenorhabditis elegans*. *DNA Repair*.

[11] Kruisselbrink E, Guryev V, Brouwer K, Pontier DB, Cuppen E, Tijsterman M (2008). Mutagenic capacity of endogenous G4 DNA underlies genome instability in FANCJ-defective *C. elegans*. *Current Biology*.

[12] Eddy J, Maizels N (2009). Selection for the G4 DNA Motif at the 5’ end of human genes. *Molecular Carcinogenesis*.

[13] Jones MR, Lohn Z, Rose AM (2011). Specialized chromosomes and their uses in *Caenorhabditis elegans*. *Methods in Cell Biology*.

[14] Rosenbluth RE, Cuddeford C, Baillie DL (1985). Mutagenesis in *Caenorhabditis elegans*. II. A spectrum of mutational events induced with 1500 r of gamma-radiation. *Genetics*.

[15] Kitao H, Hirano S, Takata M (2011). Evaluation of homologous recombinational repair in chicken B lymphoma cell line, DT40. *Methods in Cell Biology*.

[16] Sarkies P (2012). FANCJ coordinates two pathways that maintain epigenetic stability at G-quadruplex DNA. *Nucleic Acids Research*.

[17] Bridge WL, Vandenberg CJ, Franklin RJ, Hiom K (2005). The BRIP1 helicase functions independently of BRCA1 in the Fanconi anemia pathway for DNA crosslink repair. *Nature Genetics*.

[18] Deans AJ, West SC (2011). DNA interstrand crosslink repair and cancer. *Nature Reviews Cancer*.

[19] Pace P, Mosedale G, Hodskinson MR, Rosado IV, Sivasubramaniam M, Patel KJ (2010). Ku70 corrupts DNA repair in the absence of the fanconi anemia pathway. *Science*.

[20] Adamo A, Collis SJ, Adelman CA (2010). Preventing nonhomologous end joining suppresses DNA repair defects of fanconi anemia. *Molecular Cell*.

[21] Muzzini DM, Plevani P, Boulton SJ, Cassata G, Marini F (2008). *Caenorhabditis elegans* POLQ-1 and HEL-308 function in two distinct DNA interstrand cross-link repair pathways. *DNA Repair*.

[22] Wu Y, Kazuo SY, Brosh RM (2008). FANCJ helicase defective in Fanconia anemia and breast cancer unwinds G-quadruplex DNA to defend genomic stability. *Molecular and Cellular Biology*.

[23] Zhao Y, O'Neil NJ, Rose AM (2007). Poly-G/poly-C tracts in the genomes of Caenorhabditis. *BMC Genomics*.

